# ^18^F-fluoride-PET for dynamic in vivo monitoring of bone formation in multiple myeloma

**DOI:** 10.1186/s13550-016-0197-4

**Published:** 2016-05-31

**Authors:** J. C. Regelink, P. G. Raijmakers, N. Bravenboer, R. Milek, N. J. Hoetjes, A. M. de Kreuk, M. van Duin, M. J. Wondergem, P. Lips, P. Sonneveld, J. M. Zijlstra, S. Zweegman

**Affiliations:** Department of Haematology, VU University Medical Center, Amsterdam, The Netherlands; Department of Internal Medicine, Meander Medical Centre, Amersfoort, The Netherlands; Department of Radiology and Nuclear Medicine, VU University Medical Center, Amsterdam, The Netherlands; Department of Clinical Chemistry, VU University Medical Center, Amsterdam, The Netherlands; Janssen-Cilag B.V., Tilburg, The Netherlands; Department of Internal Medicine, Sint Lucas Andreas Hospital, Amsterdam, The Netherlands; Endocrine Section, Department of Internal Medicine, VU University Medical Center, Amsterdam, The Netherlands; Department of Haematology, Erasmus MC Cancer Institute, Rotterdam, The Netherlands

## Abstract

**Background:**

Bone disease in multiple myeloma is characterized by reduced bone formation. The gold standard of bone formation is the mineral apposition rate (MAR), an invasive technique reflecting bone formation at a single site. We compared ^18^F-fluoride-PET with the MAR in myeloma patients.

**Methods:**

Bone formation was measured before and after bortezomib treatment by determination of the MAR in iliac bone marrow biopsies and the measurement of ^18^F-uptake.

**Results:**

The inter- and intra-individual variations in ^18^F-uptake (SUV_A50%_) were pronounced as 33.50 (range 4.42 to 37.92) and 27.18 (range 4.00 to 31.18), respectively. A significant correlation between the MAR and ^18^F-uptake was found (*r* = 0.80, *p* = 0.017). There was a heterogeneous response after treatment varying from −2.20 to 4.53.

**Conclusions:**

Iliac ^18^F-uptake was associated with the local MAR in myeloma patients. Furthermore, ^18^F-fluoride-PET demonstrated the heterogeneity of in vivo bone formation, enabling monitoring during treatment.

**Electronic supplementary material:**

The online version of this article (doi:10.1186/s13550-016-0197-4) contains supplementary material, which is available to authorized users.

## Background

Bone disease (BD) is a major cause of morbidity and mortality in multiple myeloma (MM) patients. Up to 90 % of patients develop bone pathology throughout the course of their disease, which is not only characterized by increased osteolysis but importantly also by reduced bone formation (BF) [1]. With an increased life expectancy due to novel agents, identifying agents that in addition to their anti-MM effect improve BD is becoming more important. The proteasome inhibitor bortezomib has not only been described to inhibit osteolysis by blocking RANKL-mediated bone resorption by osteoclasts [2] but also to stimulate BF. This has been supported by the observation that the levels of biochemical markers of bone deposition increased after bortezomib treatment [3]. In addition, Giuliani showed an increase in the number of osteoblasts on the bone surface in bone marrow (BM) biopsies after bortezomib treatment [4]. However, to the best of our knowledge, in vivo data on the effect of bortezomib on BF are lacking.

One of the reasons for that is that monitoring the effect of treatment on MM-BD is hampered by the fact that the gold standard of BF, the mineral apposition rate (MAR) determined in tetracycline-labelled bone biopsies, is an invasive, laborious technique. Moreover, since MM-BD is known to have a focal and heterogeneous character with a single bone biopsy, important information will be missed. Obtaining multiple biopsies is impossible for both practical and ethical reasons. Therefore, the exploration of non-invasive, whole body techniques for the measurement of BF is of great interest. Experimental ^18^F-fluoride-PET imaging offers a potential method for the assessment of bone turnover. ^18^F-ions exchange with hydroxyl groups in the hydroxyl-apatite crystal of bone, resulting in fluoro-apatite [5]. Hence, the uptake of ^18^F reflects in vivo BF. Previous studies have shown that the quantification of ^18^F-fluoride-PET uptake had a direct relationship with local bone morphometrical changes indeed [6, 7]. Furthermore, ^18^F-fluoride-PET yields simplified quantitative parameters such as a standardized uptake value (SUV), which may be useful in the assessment of treatment response [8].

In the present study, we evaluated the accuracy of ^18^F-fluoride-PET-CT for monitoring in vivo BF by comparing ^18^F-uptake with the invasive gold standard, MAR, in MM patients being treated with bortezomib.

## Methods

This study was designed as a prospective, non-randomized single-centre pilot study. The review board approved the study, which was conducted according to the provisions of the Declaration of Helsinki, the International Conference on Harmonization, and the Guidelines for Good Clinical Practice. All patients provided written informed consent. Key inclusion criteria were bortezomib naive, relapsed MM after at least one treatment line and the presence of at least one manifest focal (osteolytic) lesion. Corticosteroid treatment in the previous 4 weeks and the presence of at least grade 2 or painful polyneuropathy were the main exclusion criteria.

Intravenous (IV) bortezomib (1.3 mg/m^2^) was administered on days 1, 4, 8 and 11 of every 21-day cycle for up to four cycles. There was no concurrent use of corticosteroids and bisphosphonates. Before treatment, ^18^F-fluoride-PET-CT was performed, with BM biopsy obtained on the first day of cycle one. These examinations were repeated at the end of the fourth treatment cycle.

Response after treatment was defined according to the International Myeloma Working Group (IMWG) response criteria.

^18^F-fluoride-PET-CT was acquired with a Gemini TF PET/CT scanner (Philips, The Netherlands). ^18^F-fluoride-PET-CT was performed 60 min post injection of 2.1 MBq/kg of ^18^F-fluoride, PET emission images were collected for 5 min per bed position from groin to skull. Low-dose CT imaging (30 mAs) was used for attenuation correction. PET-CT scans were obtained prior to the BM biopsy. Activity in the region of interest (ROI) and the volume of interest (VOI) were calculated using the standard software ROI tool (Leuven ROI tool) to define the SUV. ^18^F-uptake was measured with SUV_A50%_ [9]. The effect of bortezomib on bone remodelling was measured both visually and quantitatively per patient in predetermined bone lesions.

A control group consisting of seven healthy controls was used to validate the variability of fixation at the bone level. A comparison was made between the non-involved femoral bone of MM patients and the femoral bone of healthy individuals. In addition, the results of these seven patients were compared to the results of normal individuals as described by Puri et al. [10].

Bone biopsies of the posterior superior iliac crest were taken after tetracycline labelling. The samples were embedded in polymethylmethacrylate without prior decalcification. The histomorphometric assessment of unstained (tetracycline fluorescence) 5-μm sections was performed automatically using Nis Elements (Nikon www.nikoninstruments.com). Nomenclature was used according to the update of the American Society for Bone and Mineral Research nomenclature committee. The tetracycline double labelling of bone was used to calculate kinetic data on bone turnover. Tetracycline binds to newly formed bone at the bone/osteoid (unmineralized bone) interface where it shows as a linear fluorescence. If a second dose is given after 11–14 days, the amount of bone formed during that interval can be calculated by measuring the distance between the two fluorescent labels.

The correlation between MAR and ^18^F-uptake (SUV_A50%_) of the posterior superior iliac crest of which the BM biopsy was taken was calculated using Spearman’s correlation coefficient. A statistically significant (*p* < 0.05) correlation coefficient was used as a primary cut-off value for the acceptable accuracy for ^18^F-fluoride-PET-CT. The measurements before and after treatment were regarded as two separate outcome measures because of intermediate bortezomib treatment.

## Results

We enrolled seven consecutive patients with relapsed MM scheduled for bortezomib treatment. Two patients prematurely discontinued therapy after two cycles of therapy. As a consequence, in these patients, the MAR and PET measurements were not repeated.

**Fig. 1:**
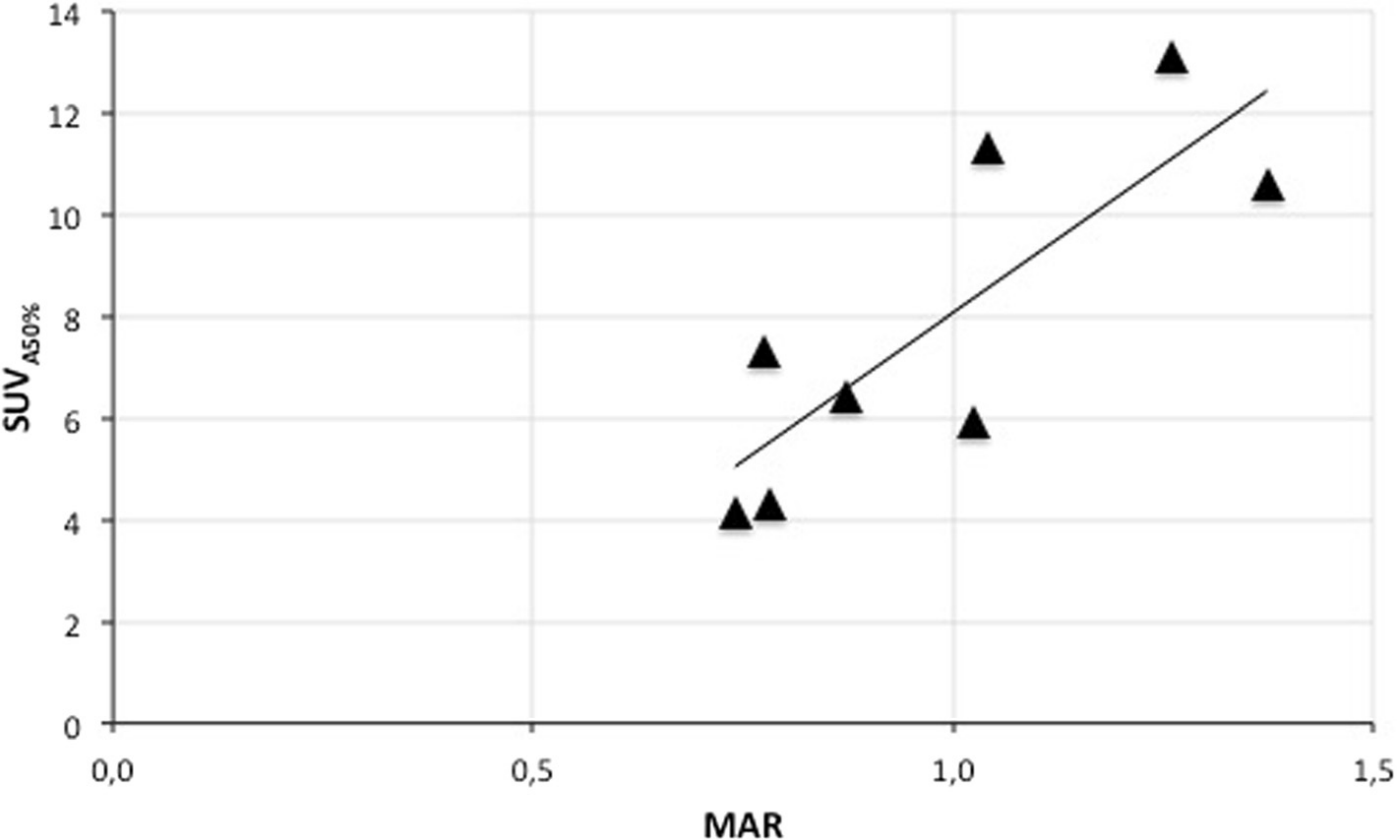
Correlation between ^18^F-fluoride-PET-CT (SUV) and mineral apposition rate

**Fig. 2:**
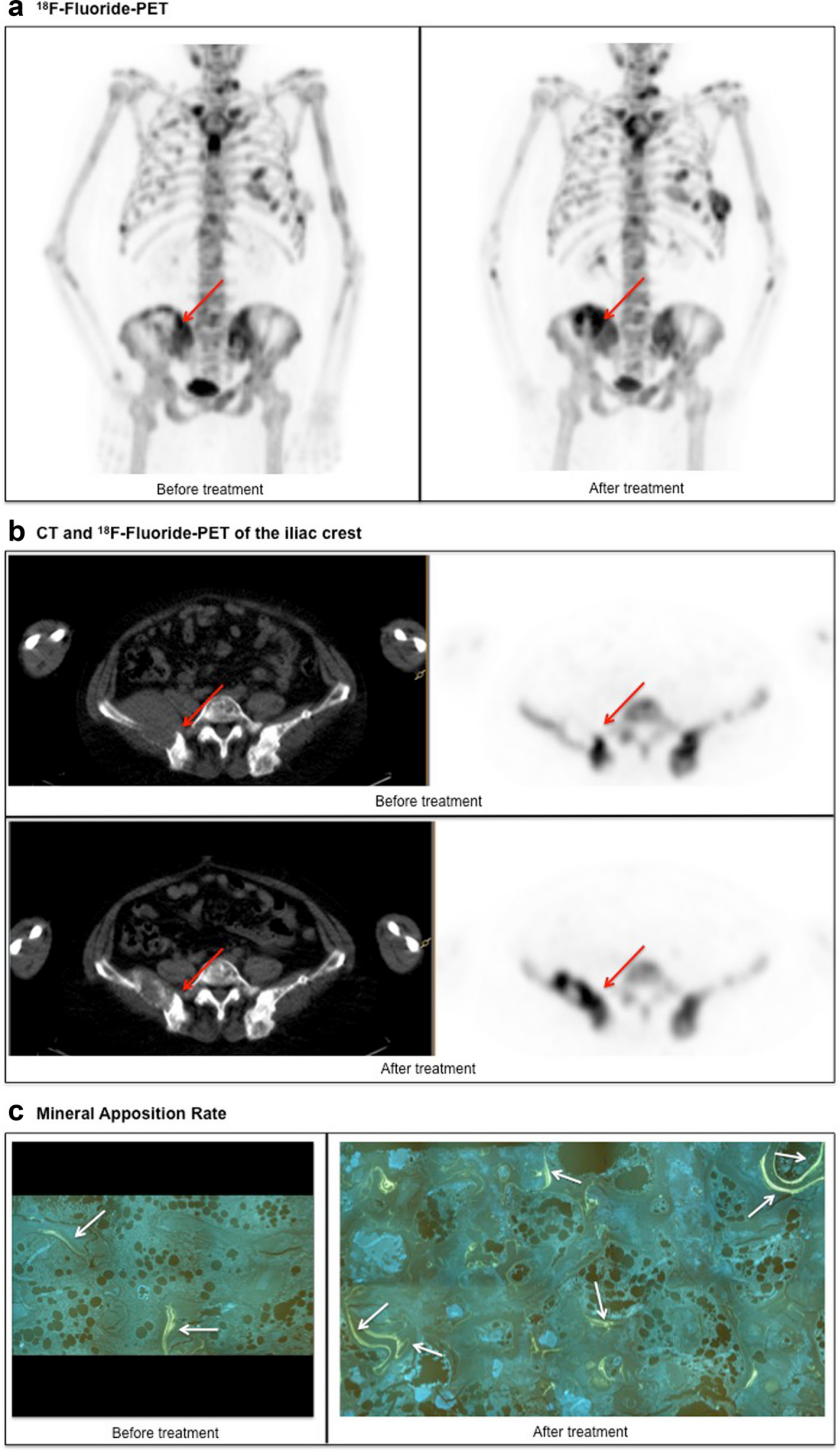
^18^F-fluoride-PET-CT and MAR (patient BBR01). **a** ^18^F-fluoride-PET before and after treatment is shown. It is illustrated that there is a large variety in ^18^F-uptake within the patient (range 7.34 to 11.34). After treatment, an increase in ^18^F-uptake in almost all lesions is seen, again with pronounced intra-individual heterogeneity (range 12.50 to 17.49). **b** CT and ^18^F-fluoride-PET of the iliac crest are shown. It is illustrated that the increase in ^18^F-uptake (11.34 to 13.09) after treatment at the site of the biopsy (*red arrow*) correlates with a decrease in extramedullary plasmacytoma and increase in osteosclerosis. **c** The biopsy was taken of the right iliac crest (*red arrow* in panel **a** and **b**). The increase in ^18^F-uptake (11.34 to 13.09) at this site is reflected by an increase in MAR (1.04 to 1.26). The double layers are pointed by *white arrows*

The MAR could be quantified in 8 of 12 biopsies. Three of the remaining four biopsies did not reveal any tetracycline labelling, and the fourth biopsy only revealed single-layer labels. In the evaluable eight biopsies, we found a significant correlation between MAR and ^18^F-uptake (SUV_A50%_) (*r* = 0.80, *p* = 0.017; Fig. 1). When considering the absence of tetracycline labelling as a lack of BF and thereby a MAR of 0, the correlation coefficient was *r* = 0.65 (*n* = 11, *p* = 0.029). An example of MAR and ^18^F-fluoride-PET-CT in an individual patient is depicted in Fig. 2.

In addition, the ^18^F-uptake in the bone of a control group consisting of seven healthy individuals, from our own database, and normal individuals as being described by Puri et al. [10] was compared. There was no difference (Mann-Whitney *U* test, *p* = 0.083), indicating that ^18^F-uptake is a reproducible method for quantification of bone remodelling indeed (Additional file 1: Table S1).

Moreover, there was no statistical difference in ^18^F-uptake (SUV_A50%_) between the *non-involved* bone (femur) of MM patients and that of healthy individuals, supporting the fact that in non-affected bone in MM patients BF is normal. (Mann-Whitney *U* test, *p* = 0.083) (Additional file 1: Table S1).

Based on conventional imaging, we identified 28 MM bone lesions in the five patients who completed therapy. There was a wide range in ^18^F-uptake (SUV_A50%_) before treatment 4.42 to 37.92. In addition, there was a pronounced intra-individual variation as well (minimum range 4.00, BBR01; maximum range 31.18, BBR05) (Table 1).

**Table 1:** ^18^F-uptake per patient before and after treatment

	^18^F-uptake (SUV_A50%_)			Disease response according to IMWG criteria
		Before treatment	After treatment	
BBR01	Minimum	7.34	12.50	Stable disease
	Maximum	11.34	17.49	
	Biopsy^a^	11.34	13.09	
BBR04	Minimum	9.19	7.09	Stable disease
	Maximum	15.74	16.00	
	Biopsy^a^	5.86	10.60	
BBR05	Minimum	6.74	6.14	Partial response
	Maximum	37.92	37.67	
	Biopsy^a^	6.42	6.19	
BBR06	Minimum	4.42	2.71	Partial response
	Maximum	16.89	17.29	
	Biopsy^a^	7.32	6.56	
BBR07	Minimum	8.86	7.13	Very good partial response
	Maximum	19.26	18.69	
	Biopsy^a^	5.91	4.31	

^a^This value represents the ^18^F-uptake at the iliac crest, which was compared with MAR. It does not represent a MM bone lesion, except in patient BBR01

**Fig. 3:**
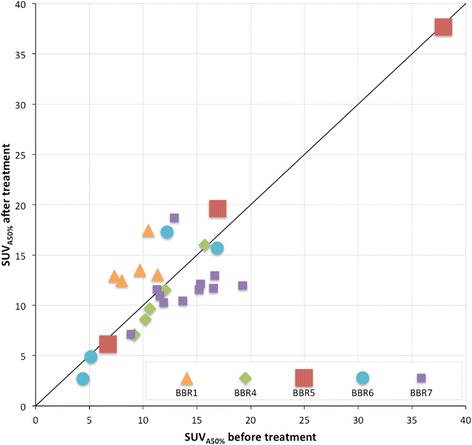
^18^F-uptake before and after bortezomib treatment. ^18^F-uptake per lesion is shown before and after treatment. It is shown that ^18^F-uptake differs between and within patients. In addition, there is a large inter- and intra-individual heterogeneity

Similar to before treatment, there was a wide range in ^18^F-uptake (SUV_A50%_) after treatment (2.71–37.67; median 11.84). Treatment did not affect ^18^F-uptake, when considering the median ^18^F-uptake of all involved lesions before and after treatment (11.74 vs 11.84, *p* = 0.61). However, a detailed analysis of individual patients revealed that the difference in ^18^F-uptake before and after treatment not only varied between patients (from −2.20 to 4.53 (median 0.48)) (Fig. 3, Table 1) but also between different lesions within the same patient. For example, in BBR04, ^18^F-uptake in the posterior iliac crest increased from 5.86 to 10.6 (ratio 1.81), with a concomitant increase in MAR from 0 to 1.37, while the BF in the MM-related bone lesions did not improve (ratios 0.77–1.02) (Fig. 3). In only one patient (BBR01), a homogeneous, statistically significant increase in ^18^F-uptake was shown due to treatment (7.34–11.34 before and 12.50–17.49 after treatment; *p* = 0.043) (Fig. 3).

## Discussion

In order to enable non-invasive in vivo monitoring of BF in MM patients, we compared the gold standard MAR with ^18^F-fluoride-PET-CT. Although we investigated a limited number of patients only, we found that ^18^F-fluoride-PET-CT, using a simplified quantitative analysis with SUV, is significantly correlated with MAR. This observation is in line with the results of Messa, who showed a correlation between bone histomorphometric indices and ^18^F-fluoride-PET in patients with renal osteodystrophy, supporting the implementation of PET for quantifying BF [3].

Moreover, we illustrated the added value of whole body imaging, by showing a large intra-individual heterogeneity in BF, thereby providing more detailed information on the regional BF as compared to MAR, reflecting BF at one site only.

Thirdly, it appears that PET can be used to monitor the effect of bortezomib therapy on BF. Earlier, an increase in osteoblastic activity after bortezomib treatment was shown by technetium (Tc) bone scintigraphy [11]. Since ^18^F-fluoride-PET-CT has a higher sensitivity and higher resolution than Tc bone scintigraphy [12], it is expected to identify more lesions. This is further supported by the fact that both patients who were described by Lee showed an increase in AP after bortezomib treatment, indicating a pronounced effect on bone remodelling, which is expected to be visualized by Tc bone scintigraphy indeed. In our patient population, AP levels were comparable before and after bortezomib treatment, whereas still changes could be observed by ^18^F-fluoride-PET-CT. Moreover, there were intra-individual changes. Therefore, ^18^F-fluoride-PET-CT is a promising technique to further unravel bortezomib-induced bone remodelling.

Thus far, there is only limited circumstantial evidence that bortezomib improves BF. Several investigators suggested an increase in BF during bortezomib treatment based on increased serum BF markers, but only Giulinani has shown an effect on patient osteoblasts. Moreover, there is debate whether this is independent from an anti-MM effect of bortezomib: although serum sclerostin (inhibiting BF) is downregulated independent of an anti-MM effect [13], the increase in the number of osteoblasts was found only in responding patients [4]. Our data, showing a pronounced inter- and intra-individual heterogeneity in BF after treatment, might explain previous controversial results in literature, as serum markers and biopsies at single sites do not reflect such anatomical diversity.

One might argue that in three biopsies no tetracycline labels were observed. This probably reflects very low or absent BF. Therefore, we defined MAR as 0. However, there might also have been a poor compliance of tetracycline intake. In order to exclude any bias, we calculated the correlation coefficient excluding the three biopsies without any label, which did not materially affect the outcome.

## Conclusions

Notwithstanding the limitations of a pilot study, the current results support the use of ^18^F-fluoride-PET-CT for the detection and evaluation of BF in MM patients. In addition, ^18^F-fluoride-PET-CT provides whole body information regarding bone remodelling. Given the pronounced intra-individual heterogeneity of bone remodelling both before and following treatment, whole body information is important and expected to be of added value to serum markers of BF to evaluate the effect of (new) treatment regimes. Our data show that ^18^F-fluoride-PET-CT instead of the gold standard MAR is an attractive technique for this evaluation.

## Additional file

Additional file 1: Table S1.
^18^F-uptake (SUV_A50%)_ in (non-involved femoral) bone of MM patients and healthy individuals. (DOCX 13 kb)
