# Supplementary material for: 18F-fluoride-PET for dynamic in vivo monitoring of bone formation in multiple myeloma
Source: EJNMMI Res. 2016 May 31;6:46. doi: 10.1186/s13550-016-0197-4 (PMC4887457; doi:10.1186/s13550-016-0197-4)
Supplement: Additional file 1: Table S1. — 18F-uptake (SUVA50%) in (non-involved femoral) bone of MM patients and healthy individuals. (DOCX 13 kb) [file 13550_2016_197_MOESM1_ESM.docx]

Additional file 1: Table S1. ^18^F-uptake (SUV_A50%)_ in (non-involved femoral) bone of MM patients and healthy individuals.

|  | MM patients | | Healthy controles |
| --- | --- | --- | --- |
|  | Before treatment | After treatment |  |
| 1 | 2.92 | 2.99 | 0.83 |
| 2 | 1.51 |  | 0.43 |
| 3 | 3.54 |  | 3,85 |
| 4 | 2.94 | 2.99 | 3,10 |
| 5 | 2.56 | 3.15 | 0.66 |
| 6 | 2.72 | 1.80 | 1.08 |
| 7 | 3.34 | 1.74 | 0.46 |
